# Inter-Residue Distance Prediction From Duet Deep Learning Models

**DOI:** 10.3389/fgene.2022.887491

**Published:** 2022-05-16

**Authors:** Huiling Zhang, Ying Huang, Zhendong Bei, Zhen Ju, Jintao Meng, Min Hao, Jingjing Zhang, Haiping Zhang, Wenhui Xi

**Affiliations:** ^1^ Shenzhen Institute of Advanced Technology, Chinese Academy of Sciences, Shenzhen, China; ^2^ University of Chinese Academy of Sciences, Beijing, China; ^3^ College of Electronic and Information Engineering, Southwest University, Chongqing, China

**Keywords:** residue distance prediction, protein structure reconstruction, deep learning, residual network, multiple sequence alignment

## Abstract

Residue distance prediction from the sequence is critical for many biological applications such as protein structure reconstruction, protein–protein interaction prediction, and protein design. However, prediction of fine-grained distances between residues with long sequence separations still remains challenging. In this study, we propose DuetDis, a method based on duet feature sets and deep residual network with squeeze-and-excitation (SE), for protein inter-residue distance prediction. DuetDis embraces the ability to learn and fuse features directly or indirectly extracted from the whole-genome/metagenomic databases and, therefore, minimize the information loss through ensembling models trained on different feature sets. We evaluate DuetDis and 11 widely used peer methods on a large-scale test set (610 proteins chains). The experimental results suggest that 1) prediction results from different feature sets show obvious differences; 2) ensembling different feature sets can improve the prediction performance; 3) high-quality multiple sequence alignment (MSA) used for both training and testing can greatly improve the prediction performance; and 4) DuetDis is more accurate than peer methods for the overall prediction, more reliable in terms of model prediction score, and more robust against shallow multiple sequence alignment (MSA).

## Introduction

Knowing the structure of a protein helps to understand the role of the protein, reveals how the protein performs its biological function, and also, sets the foundation for the protein’s interaction with other molecules. Therefore, the knowledge of a protein’s structure is very important for biology as well as for medicine and pharmacy. Since Anfinsen suggested that the advanced spatial structure of a protein is determined by its amino acid sequence (Anfinsen, 1973), it has been a “holy grail” for the computational biology community to develop an algorithm that can accurately predict a protein’s structure from its amino acid sequence. Sequence-based residue contact/distance prediction plays a crucial role in protein structure reconstruction.

Residue–residue contacts refer to the residue pairs that are close within a specific distance threshold in the three-dimensional protein structure. The contact map of a protein tells the constraints between residues in a binary form. Unlike the contact map, the distance map of a protein contains fine-grained information and, thus, provides more physical constraints of a protein structure. Protein contact/distance maps are 2D representations of the 3D protein structure and are being considered as one of the most important components in modern protein structure prediction packages. The application of predicted contacts/distances has been extended to intrinsic disorder region recognition (Schlessinger et al., 2007; Shimomura et al., 2019), protein–protein interaction prediction (Vangone and Bonvin, 2015; Du et al., 2016; Cong et al., 2019), protein design (Anishchenko et al., 2021), etc.

Contact prediction methods in the early stage are mainly based on mutual information (MI) (Pollock and Taylor, 1997; Dunn et al., 2007; Lee and Kim, 2009), integer linear programming (ILP) techniques (McAllister and Floudas, 2008; Rajgaria et al., 2009; Rajgaria et al., 2010; Wei and Floudas, 2011), traditional machine learning (ML) algorithms (Cheng and Baldi, 2007; Wu and Zhang, 2008; Tegge et al., 2009), or techniques combining ILP with ML (Wang and Xu, 2013; Zhang et al., 2016). These methods are generally considered as local strategies since a residue pair is treated statistically independent of others (Zhang et al., 2020). Breakthroughs were achieved by capturing the correlated pattern of coevolved residues by global statistical inference methods such as direct coupling analysis (DCA) (Weigt et al., 2009) and sparse inverse covariance estimation (PSICOV) (Jones et al., 2012). Methods developed based on the ideas of DCA include EVfold (mfDCA) (Morcos et al., 2011), plmDCA (Ekeberg et al., 2013), GREMLIN (Kamisetty et al., 2013), CCMpred (Seemayer et al., 2014), gDCA (Baldassi et al., 2014), and Freecontact (Kaján et al., 2014). These methods emphasize the importance of distinguishing between directly and indirectly correlated residues. Consensus-predictors like PconsC (Skwark et al., 2013), MetaPSICOV (Jones et al., 2014), and NeBcon (He et al., 2017) combine the output of different DCA-based or ML-based contact predictors to create consensus predictions. In recent years, the introduction of deep learning (DL) techniques has made tremendous progress for residue contact prediction. The DL-based contact map prediction algorithms are mainly based on convolutional neural networks (CNN) (such as DeepCov (Jones and Kandathil, 2018), DeepContact (Liu et al., 2018), and DNCON2 (Adhikari et al., 2018)), Unet [such as PconsC4 (Michel et al., 2019)], residual networks (ResNet) [such as DeepConPred2 (Ding et al., 2018), ResPRE (Li et al., 2019), MapPred (Wu et al., 2020) and TripletRes (Li et al., 2021)], ResNet combined with long short-term memory (LSTM) [such as SPOT-Contact (Hanson et al., 2018)] and transformers [such as ESM (Malinin and Gales, 2021) and SPOT-Contact-LM (Singh et al., 2022)]. COMTOP (Reza et al., 2021) uses the mixed ILP technique to combine different contact predictors (including several DL predictors) to further improve the prediction performance.

Although the predicted contacts have been successfully applied to the protein structure prediction packages (Marks et al., 2012; Michel et al., 2014; Adhikari et al., 2015; Gao et al., 2019), contact maps are still insufficient for accurate structure prediction. The reason is twofold. Most contact prediction methods use a cutoff of 8 Å between Cβ-Cβ atoms to determine whether two residues are in contact or not, resulting a contact/non-contact ratio of less than 0.1 for globular proteins and a ratio of around 0.02 for alpha-helical transmembrane proteins (Zhang et al., 2016). The definition of contacts means that the native distance information is insufficiently being distinguished. Furthermore, contact-assisted conformation sampling may be misguided by several wrongly predicted contacts and needs a long time to generate good conformations for large proteins (Xu, 2019). In this context, inter-residue distance maps are more informative than residue–residue contact maps since distances are fine-grained or real numbers, while contacts are binary values.

The methods for inter-residue distance prediction can be roughly categorized into two groups, those based on multiclass classification with discrete values and those based on regression with continuous values. Early distance maps are mainly predicted from homologous proteins (Aszódi and Taylor, 1996) or from traditional machine learning techniques (Walsh et al., 2009; Zhao and Xu, 2012; Kukic et al., 2014). The introduction of deep learning technology has injected new life into distance prediction. Wang et al. (2017) pioneered the study of introducing residual network to multiclass distance prediction. The success of this approach can be partially attributed to the ability of deep learning to simultaneously consider the global set of pair-wise interactions instead of considering only one interaction at a time, thereby leading to more accurate discrimination between direct and indirect contacts. TripletRes (Li et al., 2021), which uses a similar deep learning architecture but with a unique set of features that include multiple coevolutionary coupling matrices directly deduced from deep multiple sequence alignment (MSA) without post-processing. GANProDist (Ding and Gong, 2020) predicts real value distance as a regression problem by generative adversarial network. PDNET (Adhikari, 2020), DeepDist (Wu et al., 2021), SDP (Rahman et al., 2022), and Li et al. (2021) (Li and Xu, 2021) predict both real-valued and binned distances from residual networks. DL-based distance prediction has recently demonstrated unprecedented ability to assist protein structure reconstruction such as DMPFold (Greener et al., 2019), RaptorX (Xu, 2019), trRosetta (Yang et al., 2020), and AlhpaFold (Senior et al., 2020). However, further progress needs more accurate inter-residue distance prediction since the quality of a predicted protein structure highly depends on the accuracy of the distance prediction.

Shimomura et al. (2019) introduced a technique for predicting structurally disordered regions in proteins through average distance maps (AMD) based on statistics of average distances between residues. AMD first divides the residue pairs into different ranges according to their sequence separations, and calculates the distances of residue pairs within each range. AMD contact density maps were plotted against distance thresholds in different ranges. AMD technology detects the boundaries of structurally compact regions and finally predicts structurally disordered regions by calculating differences in density maps. The accuracy of AMD technology is comparable to the leading methods in the CASP competition such as PrDOS, DISOPRED, and Biomine. Protein domains are subunits that can fold and function independently. Therefore, correct domain boundary assignment is a critical step to achieve accurate protein structure and function analysis. Zheng et al. (2020) proposed FUPred to detect protein domains based on contact maps predicted by deep learning. The core idea of this method is to retrieve domain boundary locations by maximizing the number of intra-domain contacts while minimizing the number of inter-domain contacts from the contact map. FUpred was tested on a large-scale dataset consisting of 2,549 proteins and achieved a Matthews correlation coefficient (MCC) of 0.799 for single domain and multi-domain classification, which is 19.1% higher than the best machine learning-based method. For proteins with discontinuous domains, FUPred domain boundary detection and normalized domain overlap scores were 0.788 and 0.521, which were 17.3% and 23.8% higher than the best peer method. The results demonstrate that residue contact prediction provides a new way to accurately detect domains, especially discontinuous multi-domains. Cong et al. (2019) first compared the contact prediction methods based on mutual information, evolutionary coupling analysis, and deep learning in the prediction of residue contacts between protein complex chains and found that although the deep learning methods are outstanding for monomer contact prediction, they fail to outperform methods based on mutual information and evolutionary coupling analysis in inter-chain contact prediction. By identifying coevolving residue pairs between protein chains based on mutual information and evolutionary coupling analysis methods, 1,618 protein interactions (682 of which were unexpected) in *Escherichia coli*, and 911 protein interactions in *M. tuberculosis* (most of which were not identified in previous studies) were detected. The expected false positive rate for this study is between 10% and 20%, and the predicted interactions and networks provide a good starting point for further research. Anishchenko et al. (2021) investigated whether the residue distance information captured by deep neural networks is rich enough to generate new folded proteins. The study generated random amino acid sequences that were completely unrelated to the sequences of the native proteins used in the trRosetta training model, and fed them into the trRosetta structure prediction network to predict the starting residue distance map. Monte Carlo sampling is then performed in the amino acid sequence space to optimize the contrast between the network-predicted distribution of inter-residue distances and the background distribution averaged across all proteins. Optimization from different random starting points yields novel proteins spanning a broad range of sequences and predicted structures. Synthetic genes encoding 129 of the ‘network-hallucinated’ sequences were obtained, and the proteins were expressed and purified in *E. coli*; 27 of the proteins yielded monodisperse species with circular dichroism spectra consistent with the hallucinated structures. Three of the three-dimensional structures of the hallucinated proteins were determined by experiments, and these closely matched the hallucinated models. We can see that residue distance-assisted protein structure prediction methods can be inverted to *de novo* protein design.

In this study, we develop a method based on deep residual convolutional neural network, named DuetDis, to predict the full-length multiclass distance map from a sequence. DuetDis uses a modified ResNet module to build the network, and adopts two sets of complementary feature sets to further improve the prediction accuracy. The results by DuetDis suggest that prediction results from different feature sets show obvious differences and ensembles of different feature sets can improve the prediction performance. DuetDis is also evaluated together with 11 widely used contact/distance prediction methods, and the results show that DuetDis is more accurate for the overall prediction, more reliable in terms of model prediction score, and more robust against shallow MSA. DuetDis is available at http://hpcc.siat.ac.cn/hlzhang/DuetDis/.

## Materials and Methods

### Datasets

The test set is obtained from our previous work, containing 610 highly non-redundant protein chains (Zhang et al., 2021). The training set is obtained through culling from the whole PDB with the following criteria: 1) with maximum sequence identity of 30% against each chain in the training set and test set; 3) with structure resolutions better than 2.5 Å; 4) released before 1 May 2018 (before the beginning of CASP13). Finally, we get a non-redundant training set with 13,069 protein chains.

### Definition of Contact and Distance

In this study, the definition of contacts is directly taken from the CASP experiments. A pair of residues in the experimental structure is considered to be in contact if the distance between their Cβ atoms (Cɑ for Gly) is less than or equal to 8 Å. For direct comparison, the multiclass distance definition is taken directly from trRosetta (Adhikari, 2020). The Cβ–Cβ distance of every pair of residues in a target protein is treated as a vector of probabilities. The distance range (2–20 Å) is binned into 36 equally spaced segments, 0.5 Å each, and one bin indicating that residues are not in contact, generating a distance vector of 37 bins for each residue pair.

Depending on the separation of two residues along the sequence (*seq_sep*), the contacts are classified into four classes: all-range (*seq_sep* ≥6), short-range (6≤ *seq_sep* <12), medium-range (12≤ *seq_sep* <24), and long-range (*seq_sep* >24).

### Multiple Sequence Alignment Generation for Training and Test

**FIGURE 1:**
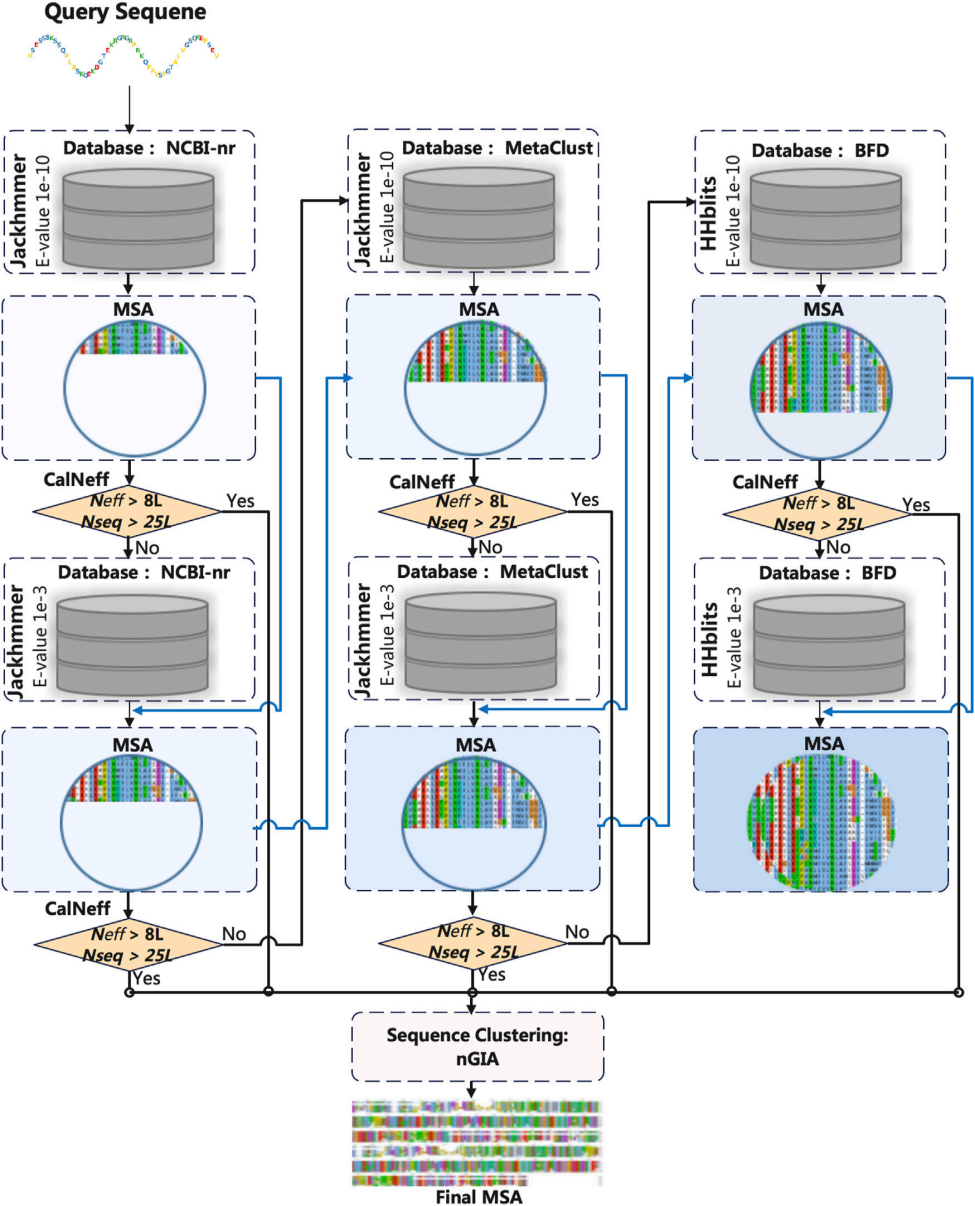
The flowchart of MSA generation for the training set.

Generating high-quality MSA is the first step for protein structure prediction based on the fact that interacting residue pairs are under evolutionary pressure to maintain the structure. The MSA used for model training is obtained as indicated in Figure 1. The target sequence in the training set is searched against NCBI-nr (Jackhmmer), MetaClust (Jackhmmer), and BFD (HHblits) respectively, with *E-*values of 1e−10 and 1e−3. The search will stop if the target MSA has *N*_*seq*_> 25*L (L is the sequence length) and *N*_*eff*_ > 8*L, where *N*_*seq*_ is the number of sequences (with sequence coverage >50%) and *N*_*eff*_ [defined in (Zhang et al., 2021)] is the number of effective sequences in the MSA. After the search, the final MSA is obtained through sequence clustering (with sequence identity of 95%) using our in-house software nGIA (Ju et al., 2021).

The MSA used for testing is obtained through searching JackHMMER (Johnson et al., 2010) against the NCBI-nr database with iteration = 3 and *E*-value = 0.0001.

### Input Features

We used two subsets of features as the inputs for the deep residual network of DuetDis. The first feature set contains 526 feature channels: one-hot-encoder of the target sequence (1D features, 20*2 channels); position-specific frequency matrix (1D features, 21*2 channels, considering gap) and positional entropy (Yang et al., 2020) (1D features, 1*2 channels); and coupling features (Yang et al., 2020) (2D features, 441 channels) derived from the inverse of the shrunk covariance matrix of MSA. The second feature set contains 151 feature channels: one-hot-encoder of the target sequence (1D features, 20*2 channels), position-specific scoring matrix (Altschul et al., 1997) (1D features; 20*2 channels; not considering gap), HMM profile (Remmert et al., 2012) (1D features, 30*2 channels), secondary structure from SPOT-1D (Hanson et al., 2019) (1D features, 3*2 channels), solvent accessible surface area from SPOT-1D (Hanson et al., 2019) (1D features, 1*2 channels), CCMPRED score (Seemayer et al., 2014) (2D features, 1 channel), mutual information (Zhang et al., 2022) (2D feature, 1 channel), and statistical pair-wise contact potential (Betancourt and Thirumalai, 1999) (2D feature, 1 channel). The first feature set, indicated as FeatSet1, is mainly composed of 2D direct coupling features (441 out of 526 total features) from the MSA, while the second feature set, indicated as FeatSet2, is mainly composed of 1D sequence-based features (148 out of 151 total features). Most of the features except the one-hot-encoder features in FeatSet1 and FeatSet2 are different, so the prediction results from the two feature sets can be complementary in a duet way (as indicated in the results).

Both FeatSet1 and FeatSet2 are widely used by previous works (Hanson et al., 2018; Yang et al., 2020; Jain et al., 2021; Su et al., 2021), showing their great efficacy in contact/distance prediction. The aim of DuetDis is not to design new feature types, but to evaluate the performance of previously widely used feature sets under the situation of unified input and identical network, as well to study how to complement the advantages of different types of features for better prediction performance.

### Deep Network Architectures and Model Training for Distance Prediction

**FIGURE 2:**
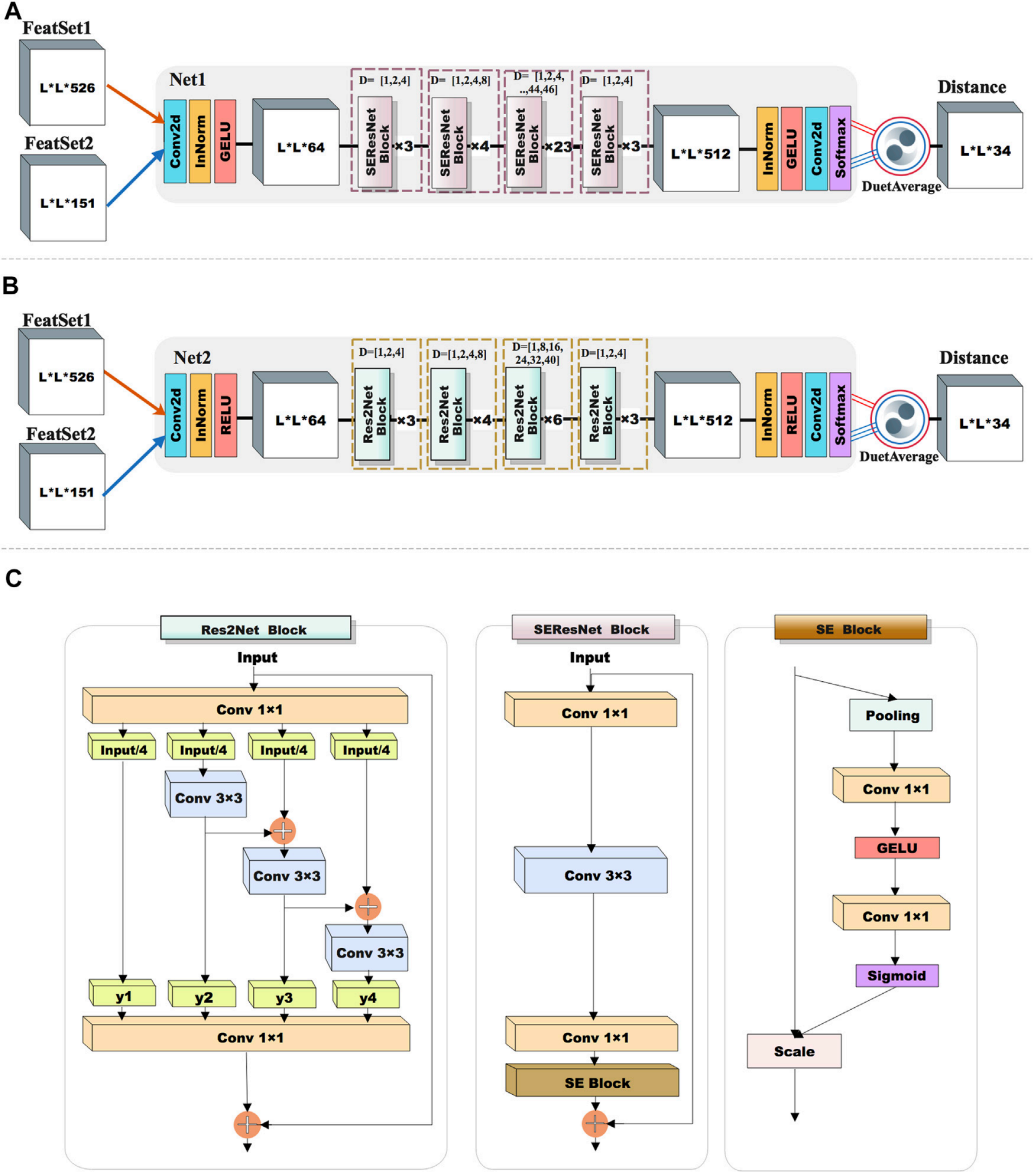
The network architecture used in this work. **(A)** The network used by DuetDis; **(B)** the reference network; **(C)** basic modules used in the networks; dilated convolution.

The proposed method DuetDis implements residual neural networks (ResNet) (He et al., 2016) as the deep learning model. Compared to traditional convolutional networks, ResNet adds feedforward neural networks to an identity map of input, which helps enable the efficient training of extremely deep neural networks. ResNet has shown its power in successful residue contact/distance prediction (Xu, 2019; Li et al., 2021). The deep residual network of DuetDis is shown in Figure 2A. The basic module of DuetDis network is a combination of squeeze-and-excitation and ResNet (SEResNet). The DuetDis network is composed of 33 SEResNet modules. In order to observe the impact of different networks and features on the prediction performance, we also designed another reference network (Figure 2B), which has very different basic modules and backbones from Figure 2A. The reference network is composed of 16 Res2Net modules. In this work, both SEResNet and Res2Net use dilation convolutions, while SEResNet use gelu and Res2Net use relu as the activation functions. The networks in Figures 2A and B are indicated as Net1 and Net2, respectively. The final MSA obtained in Figure 1 is indicated as MSA_All, and a subset with top 10 L sequences (ranked with sequence identity against the target sequence) selected from MSA_All is indicated as MSA_Top, and two disjoint subsets with each containing 10 L sequences randomly selected from MSA_All are indicated as MSA_1 and MSA_2, respectively. As described in Table 1, 10 sub-models are trained based on Net1 (the DuetDis network) and Net2 (the reference network) with different feature sets from different MSAs. “MSA Shuffle” in Table 1 means that the MSA are constructed through randomly selecting 10 L sequences in MSA_All. For each epoch, N1_M1/N2_M1 are trained through “MSA Shuffle” strategy, N1_M2/N1_M3/N2_M2/N2_M3 are trained with MSA_Top, N1_M4/N2_M4 are trained with MSA_1, and N1_M5/N2_M5 are trained with MSA_2. The outputs of five sub-models are averaged to produce the final distance map, indicated as “DuetAverage” in Figures 2A,B.

**TABLE 1:** The strategies used for the training of sub-models (N1_M1/N1_M2/N1_M3/N1_M4/N1_M5 are used for DuetDis).

Sub-models	Network	Feature set	MSA	MSA shuffle
N1_M1	Net1	FeatSet1	MSA_All	Yes
N1_M2	Net1	FeatSet1	MSA_Top	No
N1_M3	Net1	FeatSet2	MSA_Top	No
N1_M4	Net1	FeatSet2	MSA_1	No
N1_M5	Net1	FeatSet2	MSA_2	No
N2_M1	Net2	FeatSet1	MSA_All	Yes
N2_M2	Net2	FeatSet1	MSA_Top	No
N2_M3	Net2	FeatSet2	MSA_Top	No
N2_M4	Net2	FeatSet2	MSA_1	No
N2_M5	Net2	FeatSet2	MSA_2	No

The sub-models are generated by independent training branches. AdamW optimizer is performed with an initial learning rate of 0.0001 (multi-step decay is adopted as the learning rate decay strategy). Cross-entropy is used as the loss-function, and L2 regularization is used during the training process to correct overfitting. The training set is split into two parts: 600 protein chains are used as the validation set and the rest are used for training. The precision of top-L long-range contact predictions (multiclass distance map is converted to the binary contact map according to the definition in Section 2.2) on the validation dataset is calculated at each epoch, and the training process will stop when there is no update of the validation precision for 10 epochs. The training processes are implemented in Pytorch on TeslaV100 SMX2, and each independent training generally takes 5–10 days.

### Evaluation Metrics

1) The predicted distance map is a matrix of probability estimates. We analyze the performance of predictors on reduced lists of distances/contacts (sorted by the probability estimates) selected by either the probability threshold or the top-L/*n* (L is the sequence length, and *n =* 1, 2, 5) criteria. The prediction performance is assessed using precision (accuracy in some references), coverage (recall in some references), and Matthew’s Correlation Coefficient (MCC), defined as follows:

$$Precision=\frac{TP}{TP+FP},$$ (1)

$$Coverage=\frac{TP}{TP+FN},$$ (2)

$$MCC=\frac{TP\times TN-FP\times FN}{\sqrt{\left(TP+FP\right)\left(TP+FN\right)\left(TN+FP\right)\left(TN+FN\right)}},$$ (3)

where *TP*, *FP*, *TN*, and *FN* are the number of true positive, false positive, true negative, and false negative contacts, respectively.

2) Standard deviation reflects the degree of dispersion among individuals within the group, which is defined as

$$STD=\sqrt{\frac{1}{N}\overset{N}{\underset{i=1}{\sum }}{\left(x_{i}-\bar{x}\right)}^{2}},$$ (4)

where $\bar{x}$ is the mean of the variable *x*. The standard deviation can be used to evaluate the dispersion of *Precision*, *Coverage*, and *MCC.*

3) Jaccard index (Jaccard similarity coefficient) measures the similarities between sets. It is defined as the size of the intersection divided by the size of the union of two sets.

J(X,Y)=|X∩Y|/|X∪Y|, (5)

where *X* and *Y* are the set of predicted contacts from two different predictors, |X∩Y| is the number of elements in the intersection of *X* and *Y* and the |X∪Y| represents the number of elements in the union of *X* and *Y*. The Jaccard index has values in the range of [0,1], with the value of 0 for completely dissimilar ones and 1 for identical predictors.

## Results

In this section, we assess the performance of DuetDis from different perspectives. Section 3.1, 3.2 study the performance of sub-models, while Section 3.3–3.5 focus on the comparison between DuetDis and peer methods. The peer methods used in this work are 4 DCA-based contact predictors (EVfold, FreeContact, gDCA, and CCMpred), 4 DL-based contact predictors (DeepCov, PconsC4, DNCON2, and SPOT-Contact), and 3 DL-based distance predictors (TripletRes, trRosetta, and RaptorX). Section 3.1–3.3 and Section 3.5 use the results of top-L/*n* (*n* = 1, 2, 5) predictions, while Section 3.4 considers the results given by specific probability/score threshold. All sub-models and peer-methods use the same MSA as input.

### Prediction Results From Different Feature Sets Show Obvious Differences

**FIGURE 3:**
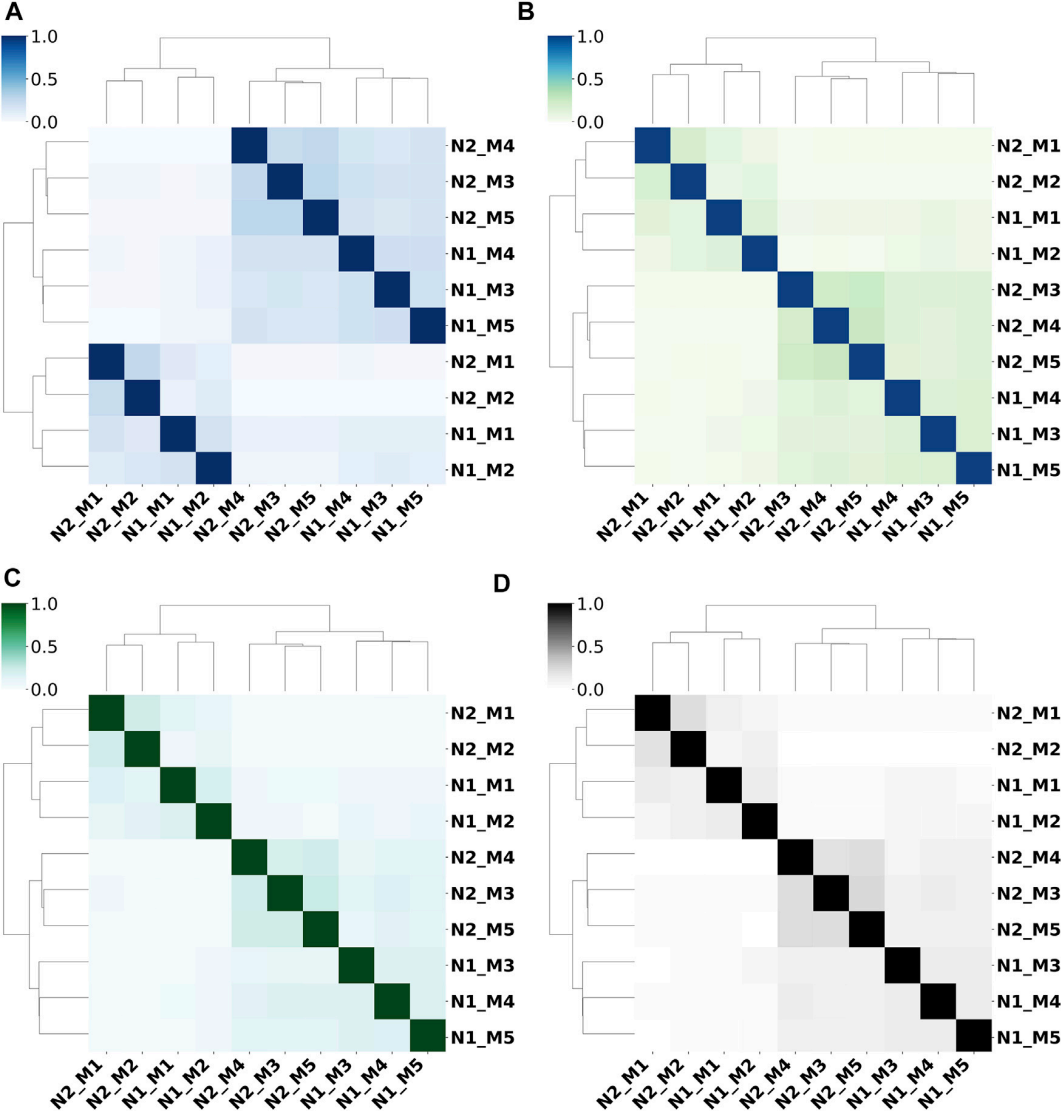
Prediction similarities between different sub-models for **(A)** all-range, **(B)** short-range, **(C)** mid-range, and **(D)** long-range contacts/distances.

We use the Jaccard indices of prediction results from 10 sub-models (as described in Table 1) to study their prediction similarities. Figure 3 shows the dendrogram heatmap of Jaccard indices using Ward’s hierarchical clustering method on the independent test set. The Jaccard index between two methods is calculated by averaging the Jaccard index value of each protein on the whole test set. According to the clustering results, these 10 sub-models can be roughly divided into two categories, and each category contains two sub-categories. N1_M1/ N1_M2 and N2_M1/ N2_M2 trained by FeatSet1 are clustered into one category (Category_1), while N1_M3/ N1_M4/ N1_M5 and N2_M3/ N2_M4/ N2_M5 trained by FeatSet2 form another category (Category_2). N1_M1/ N1_M2 trained by Net1 and N2_M1/ N2_M1 trained by Net2 form two sub-categories in Category_1, while N1_M3/ N1_M4/ N1_M5 trained by Net1 and N2_M3/ N2_M4/ N2_M5 trained by Net2 form two sub-categories in Category_2. So, we can draw the conclusion that prediction results from different feature sets show obvious differences, and the conclusion is true for all-range, short-range, mid-range, and long-range contacts/distances. The feature set decides the similarity between models for typical architectures of networks.

### Ensembling Different Feature Sets Improves Prediction Performance

The prediction accuracies of N1_M1/ N1_M2/ N1_M3/ N1_M4/ N1_M5/ N1_Ensemble (obtained by averaging the five Net1 sub-models) and N2_M1/ N2_M2/ N2_M3/ N2_M4/ N2_M5/ N2_Ensemble (obtained by averaging the five Net2 sub-models) are listed in Tables 2, 3, respectively.

**TABLE 2:** The prediction precisions of N1_M1/N1_M2/N1_M3/N1_M4/N1_M5/N1_Ensemble for different sequence separations.

Range	Method	Top-L	Top-L/2	Top-L/5
All	N1_M1	0.7769	0.8717	0.9206
	N1_M2	0.7587	0.8475	0.8941
	N1_M3	0.7491	0.846	0.9027
	N1_M4	0.7256	0.8266	0.8888
	N1_M5	0.7319	0.8328	0.8942
	N1_Ensemble	0.7896	0.8786	0.9266
Short	N1_M1	0.2955	0.481	0.7389
	N1_M2	0.2928	0.4754	0.7287
	N1_M3	0.2948	0.4757	0.7374
	N1_M4	0.2824	0.4588	0.7109
	N1_M5	0.2947	0.473	0.7219
	N1_Ensemble	0.2988	0.4918	0.7633
Medium	N1_M1	0.3512	0.5477	0.7725
	N1_M2	0.3422	0.5336	0.7514
	N1_M3	0.342	0.5329	0.7533
	N1_M4	0.3306	0.5135	0.7275
	N1_M5	0.3371	0.5209	0.7352
	N1_Ensemble	0.3537	0.5592	0.7895
Long	N1_M1	0.6245	0.7696	0.865
	N1_M2	0.6062	0.7411	0.8273
	N1_M3	0.594	0.7308	0.8246
	N1_M4	0.5695	0.7091	0.8088
	N1_M5	0.5742	0.712	0.8121
	N1_Ensemble	0.6416	0.7797	0.8626

**TABLE 3:** The prediction precisions of N2_M1/N2_M2/N2_M3/N2_M4/N2_M5/N2_Ensemble for different sequence separations. -80

Range	Method	Top-L	Top-L/2	Top-L/5
All	N2_M1	0.7532	0.8562	0.9103
	N2_M2	0.7435	0.839	0.8938
	N2_M3	0.7148	0.8188	0.8828
	N2_M4	0.7091	0.8119	0.8768
	N2_M5	0.7071	0.8121	0.879
	N2_Ensemble	0.7590	0.8579	0.9153
Short	N2_M1	0.2864	0.4654	0.7172
	N2_M2	0.2901	0.4647	0.71
	N2_M3	0.2852	0.4583	0.7014
	N2_M4	0.2831	0.4547	0.6982
	N2_M5	0.2825	0.4548	0.7002
	N2_Ensemble	0.3449	0.5396	0.7367
Medium	N2_M1	0.3413	0.5325	0.755
	N2_M2	0.3428	0.5267	0.7395
	N2_M3	0.3298	0.5082	0.7206
	N2_M4	0.3281	0.5042	0.7152
	N2_M5	0.3283	0.5057	0.7159
	N2_Ensemble	0.3449	0.5396	0.7602
Long	N2_M1	0.6035	0.746	0.8473
	N2_M2	0.5997	0.7361	0.828
	N2_M3	0.5638	0.7022	0.8066
	N2_M4	0.5525	0.6877	0.7913
	N2_M5	0.5548	0.6917	0.7941
	N2_Ensemble	0.6136	0.7508	0.8473

As we can see from Table 2, N1_M1 trained through randomly shuffling MSA_All can obtain the best performance, which is 1.8%/ 0.3%/ 0.9%/ 1.8%, 2.8%/ 0.1%/ 0.9%/ 3.0%, 5.1%/ 1%/ 2.1%/ 5.5%, and 4.5%/ 0.1%/ 2.1%/ 5% higher than N2_M2/ N2_M3/ N2_M4/ N2_M5 for top-L all-/ short-/ medium-/ long-range predictions. Although using the same network and feature set, N1_M1 shows superior prediction precisions than N1_M2, implying that randomly shuffling MSA_All in each epoch enables augmentation of the training set and thus, a better model can be obtained. N1_M3 uses the same network and feature set as N1_M4 and N1_M5, but the prediction precisions of N1_M3 are higher than N1_M4 and N1_M5, indicating that high-quality MSA used for training helps to boost the model performance. N1_Ensemble outperforms the individual sub-models N1_M1/ N1_M2/ N1_M3/ N1_M4/ N1_M5 by 1.3%/ 3.1%/ 4.0%/ 6.4%/ 5.8%, 0.3%/ 0.6%/ 0.4%/ 1.6%/ 0.4%, 0.3%/ 1.2%/ 1.2%/ 2.3%/ 1.7%, and 1.7%/ 3.5%/ 4.8%/ 7.2%/ 6.7% for top-L all-/ short-/ medium-/ long-range predictions, suggesting that ensembles of models trained on different feature sets can improve the overall prediction performance. Similar phenomenon can be observed and consistent conclusions can be drawn from the results in Table 3.

### The Overall Performance of DuetDis

**FIGURE 4:**
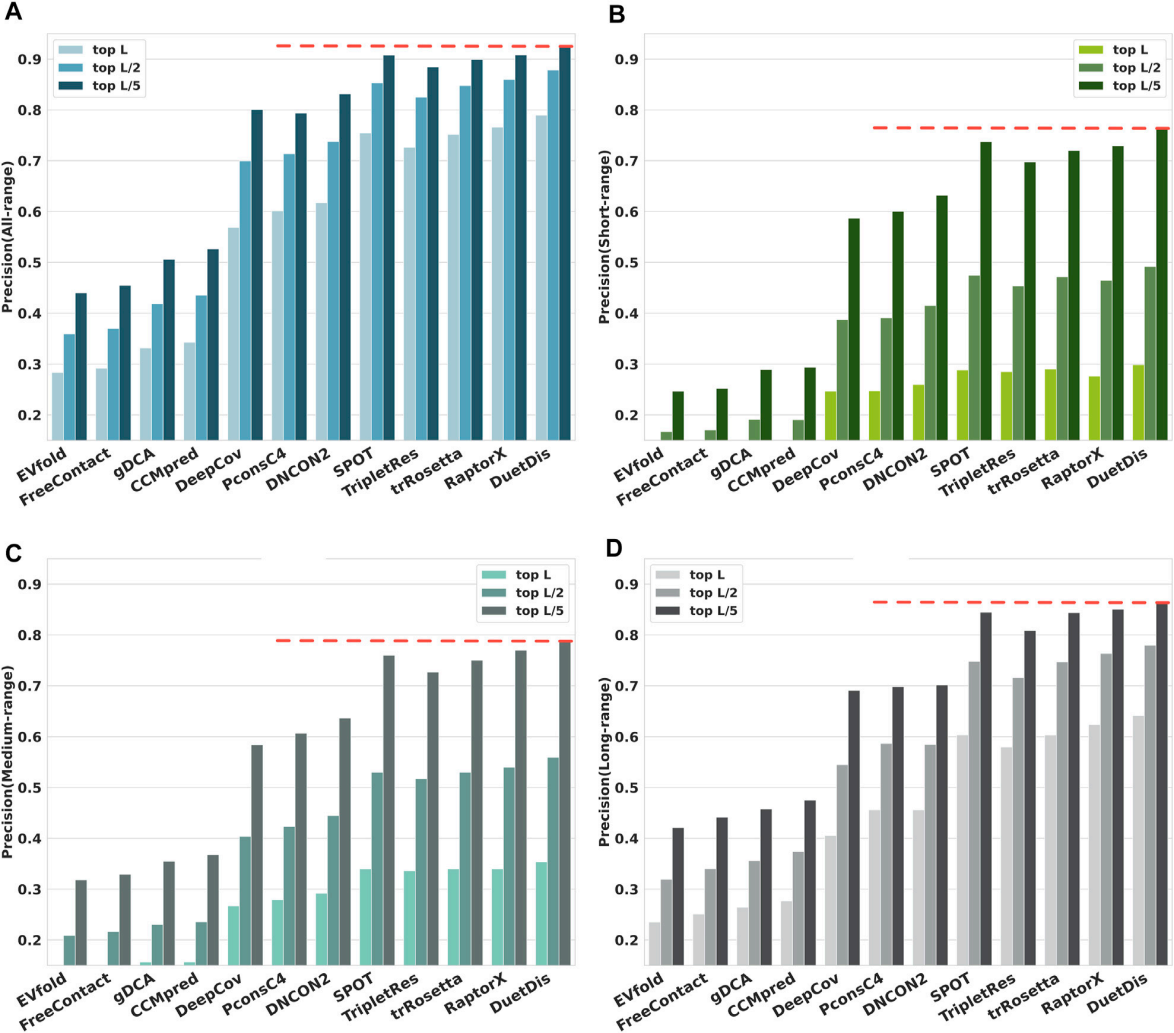
The overall prediction precisions for **(A)** all-range, **(B)** short-range, **(C)** medium-range, and **(D)** long-range contacts/distances.

The prediction precisions of all-/ short-/ medium-/ long-range contacts for DuetDis and other 11 peer methods on the independent test set are shown in Figure 4. In general, DL methods, which can capture the higher-order residue correlations and use nonlinear models with fewer parameters to be estimated from thousands of protein families (Rajgaria et al., 2010), significantly outperform DCA methods. Specifically, DuetDis shows the best overall performance. Compared with DeepCov/ PconsC4/ DNCON2/ SPOT/ TripletRes/ trRosetta/ RaptorX, DuetDis obtains 22.1%/ 18.8%/ 17.2%/ 3.5%/ 6.3%/ 3.8%/ 2.4%, 5.2%/ 5.2%/ 3.9%/ 1.0%/ 1.4%/ 0.8%/ 2.2%, 8.7%/ 7.5%/ 6.2%/ 1.4%/ 1.8%/ 1.4%/ 1.4%, and 2.4%/ 1.9%/ 3.8%/ 6.2%/ 3.8%/ 1.8% higher precisions for all-range, short-range, medium-range, and long-range top-L predictions, as well as 12.5%/ 13.3%/ 9.5%/ 1.9%/ 4.2%/ 2.7%/ 1.9%, and 17.6%/ 16.3%/ 13.1%/ 2.6%/ 6.6%/ 4.3%/ 3.4% higher precisions for all-range, short-range, medium-range, and long-range top-L/5 predictions, respectively. The better performance of DuetDis is probably due to the high-quality MSAs used for training, the delicately designed deep residual network, and the effective integration of different features.

### DuetDis Embraces High Model Reliability in Terms of Prediction Score

The confidence of the probability (score) given by a DCA or DL model can greatly reflect the reliability of the corresponding model. The prediction probabilities (scores) given by EVfold, FreeContact, gDCA, CCMpred, DeepCov, PconsC4, DNCON2, SPOT, TripletRes, trRosetta, RaptorX, and DuetDis are distributed at (0.000,1.309), (−2.537,17.931), (−1.243, 6.564), (0.000, 5.270), (0.0, 1.0), (0.0, 1.0), (0.0, 1.0), (0.0, 1.0), (0.0, 1.0), (0.0, 1.0), (0.0, 1.0), and (0.0, 1.0), respectively. For machine learning (both traditional and deep learning) applications, people usually use 0.5 as a threshold for classification. However, the threshold may be inaccurate for a complex problem like contact/distance prediction. Therefore, studying the scoring trend and the reliability of the model is of great benefit to understand the model performance.

**FIGURE 5:**
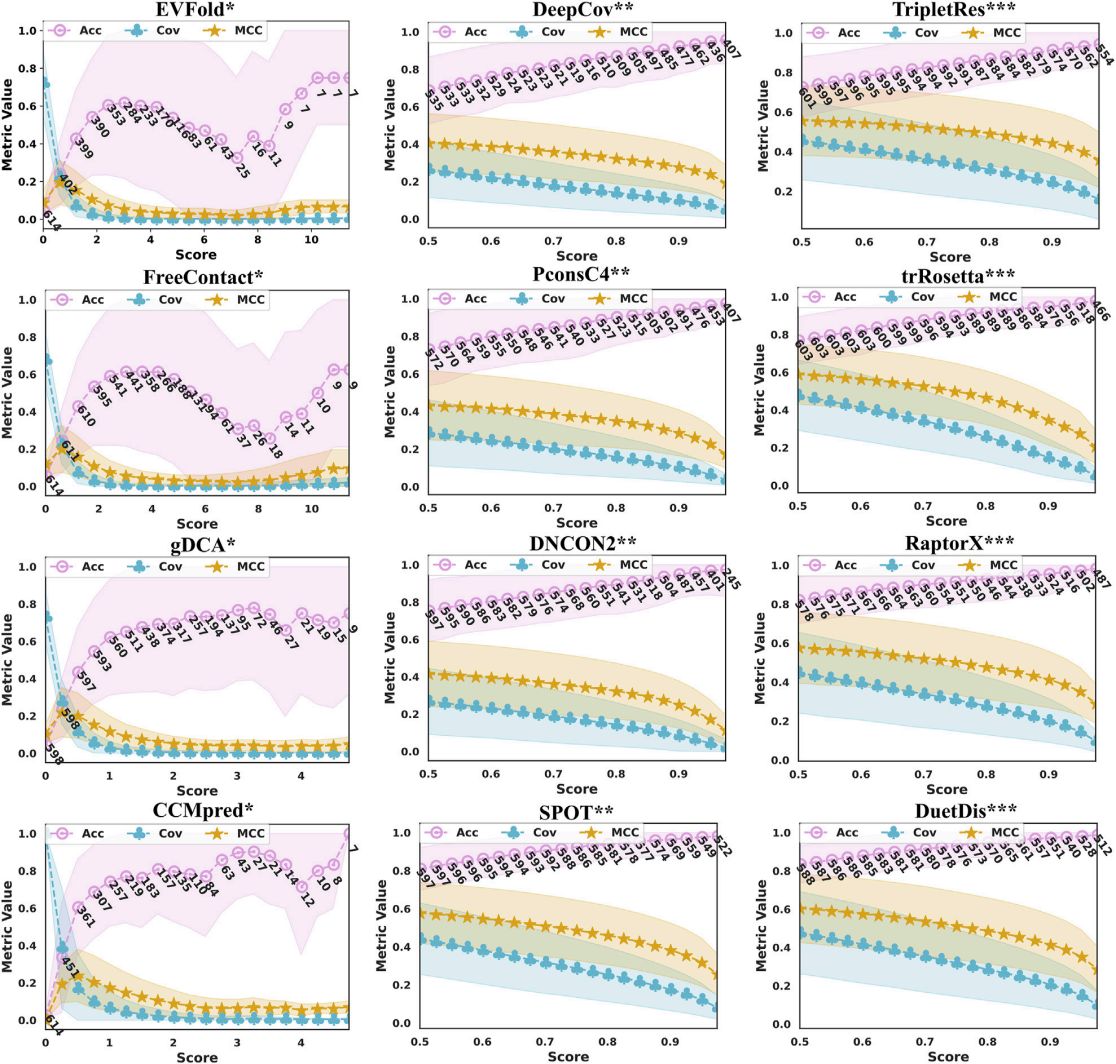
Prediction performance in terms of precision, coverage, MCC, and the corresponding standard deviation (the shaded area around the curves) with the increasing probability (score) threshold given by the predictors. The numbers under the precision curve (blue) are the numbers of proteins with predictions returned using the corresponding (score) threshold on the x-axis.

Figure 5 illustrates the prediction performance in terms of precision/ coverage/ MCC with the increase in probability (score) threshold given by DuetDis and the peer methods. With the increase of the probability (score) threshold, the prediction coverages decrease monotonically for all methods. As the threshold increases, their precision curves go down at some probability (score) value. The prediction precisions of all DL methods (DeepCov/ PconsC4/ DNCON2/ SPOT/ TripletRes/ trRosetta/ RaptorX) increase monotonically with the probability (score) threshold. However, the precision curves of DCA methods (EVfold/ FreeContact/ gDCA/ CCMpred) show turning points at some probability (score) values. Meanwhile, DCA methods also show much larger STDs on precisions and relatively lower coverages/MCCs compared with DL methods. The numbers under the precision curve in Figure 4 are the numbers of proteins with predictions returned using the corresponding probability (score) threshold on the x-axis. It is obvious that, as the probability (score) threshold increases, there are more proteins being predicted by DL methods than by DCA methods. Specifically, DuetDis achieves prediction precisions/ coverages/ MCCs of 98.1%/ 15.0%/ 0.352 (calculated on the 523 proteins with prediction scores higher than 0.95) at the (score) threshold of 0.95, which are higher than that by DeepCov (94.7%/ 7.4%/ 0.240: 431 proteins), PconsC4 (96.3%/ 6.5%/ 0.228: 448 proteins), DNCON2 (96.8%/ 4.4%/ 0.173: 396 proteins), SPOT (97.5%/ 12.3%/ 0.318: 544 proteins), TripletRes (93.0%/ 19.6%/ 0.399: 557 proteins), trRosetta (96.5%/ 9.4%/ 0.276: 513 proteins), and RaptorX (97.2%/ 14.5%/ 0.352: 497 proteins). In summary, DuetDis shows higher reliability in model probability (score) compared with peer methods.

### DuetDis Is Robust Against Shallow Multiple Sequence Alignment

**FIGURE 6:**
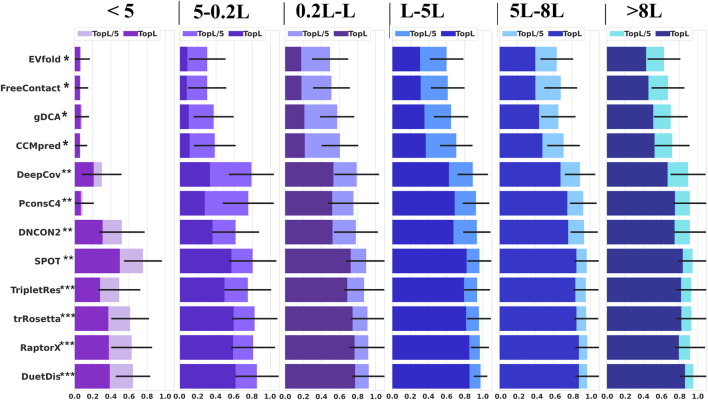
Prediction precisions of different methods for all-range, top-L, and top-L/5 predictions with the variation of *N*_*eff*_. The error bar is the standard deviation of all precisions (for top L/5 predictions) in each sub-test set.

Coevolutionary coupling signals extracted from MSA play central role in most modern contact/distance prediction methods. In this study, the independent test set is divided into six groups according to *N*_*eff*_ (<5, 5–0.2 L, 0.2 L–L, L–5 L, 5–8 L, and >8 L). The performance of different methods on these sub-groups of the test set is shown in Figure 6. DuetDis achieves prediction precisions of 64.4% for *N*_*eff*_ <5, 85.1% for *N*_*ef*_ = 5–0.2 L (2.5% higher than the second), 92.5% for *N*_*eff*_ = 0.2 L–L (0.5% higher than the second), 97.5% for *N*_*eff*_ = L–5 L (0.8% higher than the second), 96.9% for *N*_*eff*_ = 5–8 L (0.2% higher than the second), and 95.6% for *N*_*eff*_ = 5–8 L (0.9% higher than the second). For *N*_*eff*_ <5 L, DuetDis ranks the second in prediction precision; while for *N*_*eff*_ = 5–0.2 L, 0.2 L–L, L–5 L, 5–8 L and >8 L, DuetDis is in the leading position of prediction precision. For Neff <5 L, PconsC4 shows a STD of 0.125 which is smaller than DuetDis, however, the smaller STD is because of lower overall precision by PconsC4 (the average prediction precisions are 8.7% for PconsC4 and 64.4% for DuetDis). Hence, DuetDis obtains the least STD among all DL methods for all sub-groups of the test set. In general, DuetDis shows leading precisions and the smallest STD for most ranges of *N*_*eff*_, especially highlights its robustness in shallow MSA-based distance prediction.

## Conclusion

Proteins are considered as the molecular machines and perform many important functions of life (Zhang et al., 2017). Knowing the structure of a protein helps to understand the role of the protein, how the protein performs its biological function, and the interaction between the protein and the protein (or other molecules), which is very important for biology as well as for medicine and pharmacy. Residue distance prediction from the sequence is critical for many biological applications such as protein structure reconstruction. However, prediction of large distances and distances between residues with long sequence separation length still remains challenging.

In this paper, we propose DuetDis, which uses duet deep learning models for distance prediction. DuetDis adopts two complementary feature sets, one set is mainly composed of 2D coevolutionary couplings, and another set contains mainly 1D sequence-based features. We trained 10 sub-models using two different networks (Net1 and Net2), two different sets of features (FeatSet1 and FeatSet2), and four different MSAs (MSA_All, MSA_Top, MSA_1, MSA_2). By evaluating 10 sub-models based on the large-scale test set, we found that: 1) prediction results from different feature sets show obvious differences; 2) ensembling different feature sets can improve the prediction performance; and 3) high-quality MSA used for both training and testing can greatly improve the prediction performance. DuetDis is also compared with 11 widely used contact/distance predictors. The experimental results show that DuetDis outperforms the peer methods in terms of overall prediction precisions, model reliability, and robustness against shallow MSA.

## Data Availability

The original contributions presented in the study are included in the article, further inquiries can be directed to the corresponding author.
